# Genome-Wide Analysis of the GRF-GIF Module in *Coffea arabica* L.: Insights into the Starlet-Flower Phenomenon

**DOI:** 10.3390/ijms27136007

**Published:** 2026-07-04

**Authors:** Gabriel de Campos Rume, Raphael Ricon de Oliveira, Isabel Marques, Antonio Chalfun-Junior, José Cochicho Ramalho

**Affiliations:** 1 Laboratory of Plant Molecular Physiology, Plant Physiology Sector, Department of Biology, Institute of Natural Sciences, Federal University of Lavras (UFLA), Lavras 37200-900, MG, Brazil; gabriel.rume@gmail.com; 2 Department of Biological Sciences, State University of Santa Cruz (UESC), Ilhéus 45662-900, BA, Brazil; rapharicon@gmail.com; 3 Forest Research Centre (CEF), Associate Laboratory TERRA, School of Agriculture (ISA), University of Lisbon (ULisboa), Tapada da Ajuda, 1349-017 Lisbon, Portugal; isabelmarques@isa.ulisboa.pt (I.M.); cochichor@isa.ulisboa.pt (J.C.R.)

**Keywords:** abiotic stress response, floral organogenesis, phylogenetic analysis, RNA-seq, RT-qPCR, transcription factor

## Abstract

The Growth-Regulating Factor (*GRF*) family and their co-activators, GRF-Interacting Factors (*GIFs*), are key players in the trade-off between plant development and stress adaptation, functioning as canonical targets of the highly conserved miR396 family, which mediates responses to environmental stressors including high temperatures and water deficits. The “starlet” phenomenon in the allotetraploid *Coffea arabica* L. is a developmental disorder that results in malformed flowers, frequently associated with environmental stress and floral sterility. Since their underlying molecular mechanisms remain uncharacterized, we performed a genome-wide identification of the *CaGRF* and *CaGIF* families and quantified their transcriptional profiles in shoot apical meristems (SAMs) and across multiple stages of floral bud development. Our findings reveal significant differential expression of the GRF-GIF module between typical and starlet tissues throughout development, including the SAM. Intriguingly, these results do not correlate with the levels of a representative member of the miR396 family, indicating that the GRF-GIF expression shifts in starlet-flowers may be uncoupled from miR396 levels. This work provides the first molecular insights into the enigmatic starlet phenomenon in *Coffea arabica* L., addressing an understudied aspect of coffee reproductive development and its implications for the reproductive stability (and productivity) of this important tropical crop species.

## 1. Introduction

As key players in the trade-off between plant development and stress adaptation, Growth-Regulating Factors (*GRFs*) comprise a plant-specific transcription factor (TF) family defined by two highly conserved N-terminal domains: the QLQ (Glutamine, Leucine, Glutamine) and WRC (Tryptophan, Arginine, Cysteine) motifs [1]. The QLQ domain facilitates protein-protein interactions, while the WRC domain contains a nuclear localization signal and a zinc-finger motif required for DNA binding [1,2]. Family members typically exhibit overlapping functions and are strongly expressed in young, developing tissues, where they play a role in the maintenance of the proliferative capacity of primordial cells by regulating the expression of genes related to the mitotic cycle, such as Knotted1-Like Homeobox (*KNOX*); thus, they control the fine balance between the maintenance of meristematic activity and the onset of organ differentiation [1,3,4]. GRFs are canonical targets of the highly conserved miR396 family, known to be involved in various stress responses which include high temperatures and water deficits [5,6,7,8,9], conditions frequently associated with the starlet-flower phenomenon of *Coffea arabica* L. This microRNA post-transcriptionally suppresses GRF expression through mRNA cleavage or translational inhibition [10], implicating the module as a pivotal regulator in the trade-off between growth processes and stress responses. Numerous studies have demonstrated an inverse correlation between the expression of miR396 and its targets and have evaluated the role of the miR396-GRF module in plant development [2,3,11]. Given its role in determining the final size, shape, and longevity of the floral organs, disruptions in this module have been shown to induce floral abnormalities and reduced fertility in model and crop species, such as *Arabidopsis thaliana* (L.) Heynh., *Nicotiana tabacum* L., and *Oryza sativa* L. [12,13,14], indicating the importance of *GRF* expression for floral organogenesis.

To regulate plant growth, GRFs recruit coactivators from the ancient GRF-Interacting Factor (*GIF*) family, characterized by a conserved N-terminal SNH (SYT N-terminal homology) domain [2,15,16]. This SNH domain physically interacts with the QLQ domain of GRF proteins to form an obligatory hetero-complex, required for sustained cell proliferation and coordinated organogenesis [17]. This association likely emerged early in the evolution of Streptophyta, as the QLQ domain is present in plant and human BRAHMA (BRM) homologs, ATPases central to the evolutionarily conserved Switch/Sucrose Nonfermenting (SWI/SNF) chromatin-remodeling complexes [2,18]. Accordingly, *A. thaliana* GIF1/ANGUSTIFOLIA3 (AN3) recruits these complexes to increase promoter accessibility, thereby enhancing the transcriptional regulation of target genes and the auto-regulation of the *GRF* family itself [2,19]. The biological significance of this module is evident in *A. thaliana gif1/an3* mutants, which exhibit restricted cell proliferation in leaves and petals, while *gif* triple mutants show impaired inner floral whorl development and gametogenesis, leading to reduced fertility or complete sterility [13,20,21,22]. However, the observation that neither *Arabidopsis* nor maize GIF1 interact with all available GRFs indicates that module function may be dependent on the specific composition of the GRF-GIF complex [16,17,21,23,24]. Consequently, the biological outcome is contingent on the overlapping spatiotemporal expression patterns of individual members.

Coffee is one of the most widely consumed beverages globally, reaching approximately one third of the world’s population [25]. Within the *Coffea* genus, *C. arabica* constitutes the most important cultivated species, accounting for ca. 57% of the world’s production in the 2024 coffee year [26]. *C. arabica* is the only polyploid species of the genus, characterized as an allotetraploid (2n = 4x = 44) originating from the hybridization of the unreduced gametes of *C. canephora* and *C. eugenioides* [27,28]. Coffee cultivation is notably affected by the climate, with studies suggesting that projected increases in temperature, coupled with global-scale changes in precipitation patterns, could severely affect both cultivated and wild species, potentially leading to declines in productivity and habitat suitability and even wild species extinction with significant losses of genetic diversity [29,30]. However, the actual severity may be lower than early models predicted, as elevated [CO_2_] can mitigate environmental stress and improve the metabolic and reproductive performance of *Coffea* spp. [31,32,33].

A developmental abnormality of the allopolyploid is the emergence of malformed flowers, apparently with altered fertilization ability, characterized by their stunted organs and premature anthesis [34]. Named for their characteristic star shape, these starlet-flowers differ from the typical white, expanded coffee flowers by instead exhibiting atrophied green petals [35]. The phenotype’s intensity varies significantly: affected plants may exhibit exclusively anomalous flowers or a stochastic mosaic. In the latter, the starlet-flowers are interspersed with normal ones, occurring both within the same floral node and heterogeneously across different nodes and branches of the tree. Some authors have associated the emergence of these starlet-flowers with stress exposure, namely prolonged periods of high temperature and/or water deficit occurring during the early stages of floral bud development [25,34,36]. However, despite the frequent observation of such disorder in the field, little has been explored about the phenomenon to this date.

In this study, we conducted genome-wide identification and characterization of the *CaGRF* and *CaGIF* families within the *C. arabica* genome. We compared the expression patterns of these important genes, alongside a representative miR396 family member, in shoot apical meristems (SAMs) and across multiple stages of floral bud development in plants producing both normal and starlet-flowers. To the best of our knowledge, this work represents the first molecular analysis to provide insights into the emergence of the starlet-flowers, an understudied phenomenon in coffee reproductive development. In fact, unveiling the genetic factors governing floral development is crucial to understand the reproductive potential of this important crop under changing environmental conditions, especially under the likely intensification of climate stressors associated with high temperature and lower water availability.

## 2. Results

### 2.1. In Silico Identification and Characterization of the GRF and GIF Families in C. arabica

A total of 15 GRF and 6 GIF members were identified and named *CaGRF1* to *CaGRF15* and *CaGIF1* to *CaGIF6*, respectively (Table S1). This nomenclature was established according to their physical order of appearance along the coffee plant’s genome, following a logical numerical progression from chromosome 1 to 11 and between the homeologous subgenomes C (from *C. canephora*) and E (from *C. eugenioides*). Multiple sequence alignments confirmed the presence of the QLQ and WRC domains across all CaGRFs, while the conserved N-terminal SNH domain was identified in every CaGIF sequence, supporting their classification as canonical members of the GRF and GIF families (Figure S1). Notably, CaGRF2 and CaGRF3 feature an additional, truncated WRC domain situated in the C-terminal region, distinguishing them from all other identified proteins. Furthermore, *CaGRF4*, *CaGRF5*, and *CaGRF6* encode identical protein sequences.

**Figure 1:**
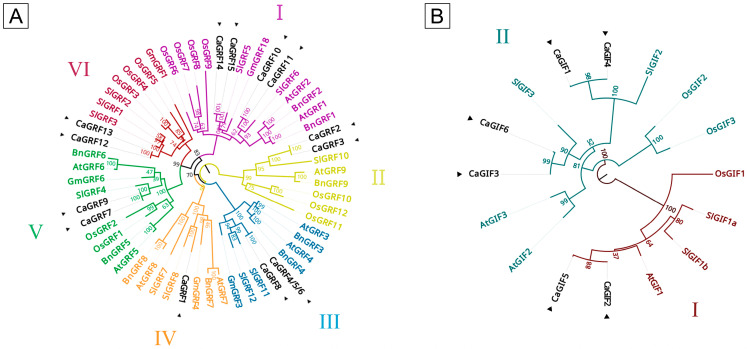
Phylogenetic trees of CaGRF and CaGIF sequences. (**A**) CaGRF and (**B**) CaGIF sequences identified in *C. arabica* (Ca, indicated by black triangles) are displayed alongside homologs from *A. thaliana* (At), *G. max* (Gm), *O. sativa* (Os), and *S. lycopersicum* (Sl). Members are distributed among each family’s established subgroups (indicated around the trees). Node values represent the bootstrap support derived from 1000 replicates. Due to absolute sequence identity, CaGRF4, CaGRF5, and CaGRF6 were merged into a single branch labeled as CaGRF4/5/6. Additional details are available in Table S1.

To classify the sequences within established subgroups of the GRF and GIF families, separate phylogenetic trees were constructed using CaGRFs and CaGIFs alongside reference sequences (Figure 1). Due to the absolute sequence identity shared among CaGRF4, CaGRF5, and CaGRF6, these proteins were merged and are denoted as CaGRF4/5/6 within the phylogenetic tree. The high bootstrap support observed across the trees enabled the identification of homologous relationships and putative orthologues in model species, such as *A. thaliana*, *O. sativa*, and *Solanum lycopersicum* L., allowing tentative functional inferences based on previously characterized genes.

The inspection of the phylogenetic tree comprising the GRF sequences enabled the classification of at least one coffee protein into each of the established subgroups. Among the fifteen sequences, one was assigned to group IV, while two were placed in groups II, V, and VI and four were clustered within groups I and III. Likewise, the analysis of the GIF sequences indicated their distribution into the two distinct clades of the transcriptional coactivator family, with two sequences positioned in clade I and four in clade II.

The exon–intron organizations and conserved protein motifs of both gene families further support the phylogenetic classifications. Gene structure analysis revealed that all genes from both families harbor four exons and three to four introns, with distribution patterns that are highly conserved among members of the same phylogenetic clades (Figure S2). This high degree of conservation was particularly evident between homeologous gene pairs, which exhibited nearly identical structural organizations. Additionally, motif elicitation analysis identified distinct conserved motifs beyond the canonical domains. Consistent with the gene structure patterns, the composition and arrangement of these motifs exhibit variations between different subfamilies for both CaGRFs (Figure S3) and CaGIFs (Figure S4). Notably, CaGRF1 possesses only the structural minimum required to be classified as a GRF, specifically, the canonical QLQ and WRC domains, while lacking the additional conserved motifs present in the sequences of other family members.

Analysis of the promoter regions of *CaGRFs* and *CaGIFs* revealed a diverse array of putative *cis*-regulatory elements (CREs, Figure S5), displaying highly similar distribution profiles between homeologous pairs. In both gene families, in addition to core promoter elements required for transcription, such as the CAAT-box and TATA-box, a significant number of elements responsive to light responsiveness and phytohormone signaling were also identified. Specifically, elements associated with auxin (AACGAC and GGTCCAT) abscisic acid (ABRE), gibberellins (GARE and P-box), salicylic acid (TCA), and methyl jasmonate (CGTCA and TGACG) were notably abundant. Furthermore, promoters of *CaGRFs* also featured elements linked to abiotic stressors, including low temperature, drought and anaerobiosis. The prediction of CREs also revealed elements associated with seed development. The RY motif, potentially involved in seed-specific regulation, was identified in the upstream regions of *CaGRF4*, *CaGRF5*, *CaGRF6*, and *CaGRF8*, encompassing all members of clade III, while another motif related to seeds, GCN4, was identified in *CaGRF7* and *CaGRF9* (clade V), *CaGRF12* and *CaGRF13* (clade VI), and *CaGRF14*, in addition to *CaGIF2* and *CaGIF5*, the sole members of clade I within the coactivator family.

### 2.2. CaGRFs and CaGIFs Are Similarly Positioned Within the Remarkably Collinear Subgenomes of C. arabica

**Figure 2:**
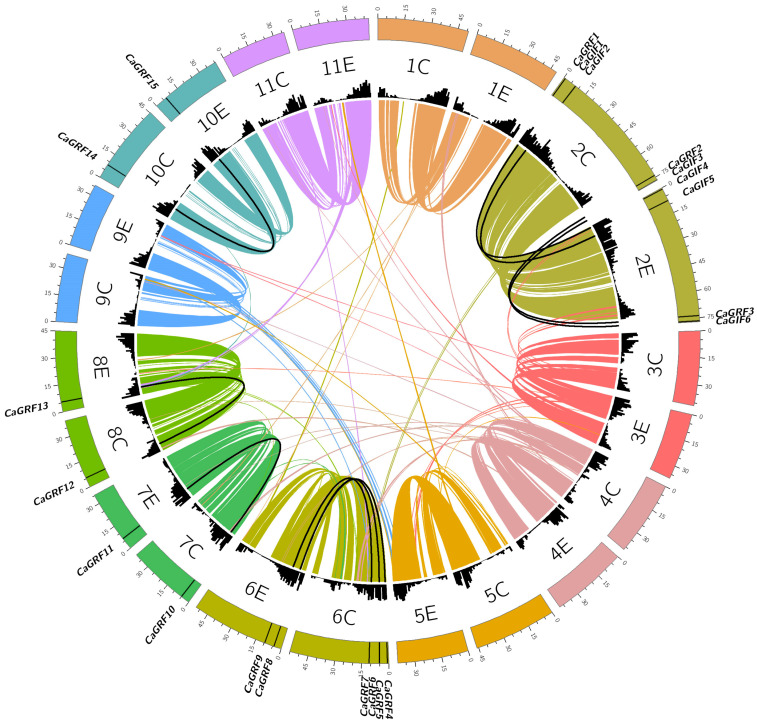
Chromosomal distribution and syntenic relationship of *CaGRF* and *CaGIF* families in the *C. arabica* genome. The fifteen *CaGRFs* and six *CaGIFs* are mapped across the 11 chromosome pairs of the allotetraploid genome. Syntenic relationships between the two subgenomes (C and E, representing the ancestral *C. canephora* and *C. eugenioides* subgenomes, respectively) are indicated by colored lines. Black lines highlight syntenic links between identified *CaGRFs* or *CaGIFs*. The black histogram track depicts the density of syntenic links across the genome, reflecting regions of high collinearity. Gene coordinates can be estimated using the scale (Mb) situated on the outer track of each chromosome.

A chromosomal map was constructed to visually depict the distribution of identified genes across the *C. arabica* genome (Figure 2). Noticeably, all *CaGIFs* were localized on the second chromosome pair, whereas *CaGRFs* were more broadly distributed throughout the genome, occurring on five of the eleven chromosome pairs (chromosome pairs 2, 6, 7, 8 and 10). The self-synteny analysis, which identifies conserved gene blocks duplicated within the same genome, evidenced the pronounced collinearity between the two subgenomes that constitute the allotetraploid (Figure 2). Syntenic link density was higher in distal regions of one or both chromosome arms, suggesting greater conservation between subgenomes in these regions compared to proximal segments. All identified genes were located within these highly syntenic regions. While most have a corresponding homeolog, *CaGRF1* appears to be unique to the *C. canephora* subgenome.

### 2.3. Transcriptional Profiling Reveals Distinct Spatiotemporal Dynamics Within the CaGRF and CaGIF Families

**Figure 3:**
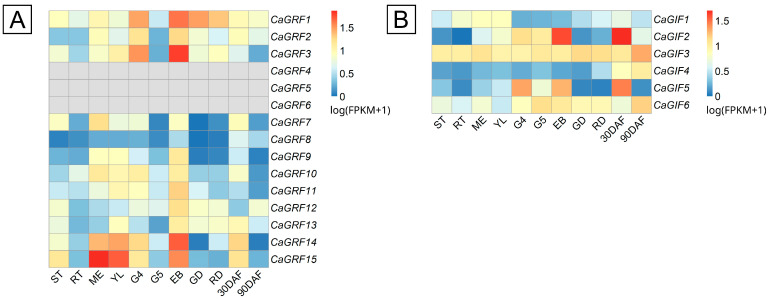
Spatiotemporal expression patterns of (**A**) *CaGRFs* and (**B**) *CaGIFs*. Heatmaps illustrate the relative transcript abundance across diverse tissues and developmental stages. Color intensity represents expression levels calculated as log_10_(FPKM + 1), ranging from low (blue) to high (red) relative expression. Gene identifiers are indicated on the right of each panel, whereas the tissues are labeled at the base: ST, stems; RT, roots; ME, meristems; YL, young leaves; G4 and G5, floral buds at developmental stages G4 and G5; EB, embryos; GD, green drupes; RD, red drupes; 30DAF and 90DAF, fruit perisperm at 30 and 90 days after flowering, respectively. FPKM: Fragments Per Kilobase of transcript per Million mapped reads.

To validate the transcriptional activity of the identified *CaGRFs* and *CaGIFs*, expression profiles were generated using transcriptome data from 17 publicly available RNA-seq libraries. Tissues analyzed comprise stems (ST), roots (RT), meristems (ME), young leaves (YL), embryos (EB), floral buds (G4 and G5) and fruits at different developmental stages (GD, RD, 30DAF and 90DAF; Figure 3). Accession numbers and further information on the respective libraries are provided in Table S1.

Distinct expression profiles were observed between groups of the identified genes. Notably, genes grouped within the same phylogenetic clades displayed similar expression patterns. Specifically, *CaGRF4*, *CaGRF5* and *CaGRF6*, which encode identical proteins and are positioned within clade III, displayed undetectable transcript counts, while *CaGRF8*, the remaining member of this clade, showed very low transcript abundance relative to the other family members. In contrast, *CaGRF1*, *CaGRF3*, *CaGRF14*, and *CaGRF15* were the most abundant transcripts, particularly in actively growing tissues, exhibiting distinct spatiotemporal profiles. Specifically, *CaGRF14* and *CaGRF15* were the predominant transcripts in meristems and young leaves, whereas *CaGRF1* and *CaGRF3* showed the highest expression levels in flower buds. Notably, all four of these genes displayed prominent expression in embryos. During fruit development, *CaGRF1* maintained high expression across green and red drupes, as well as in the perisperm at 30 and 90 days after flowering (DAF). *CaGRF14* and *CaGRF15* also emerged as major transcripts in the 30 DAF perisperm (Figure 3A).

Regarding the *CaGIF* family, *CaGIF3* exhibited stable and relatively high expression levels across all analyzed libraries, whereas *CaGIF6* showed high expression across most of them (Figure 3B). In contrast, *CaGIF1* and *CaGIF4* showed comparatively lower and more tissue-specific transcript abundance. Interestingly, although these two genes constitute a homeologous pair within clade II, their expression patterns differed greatly: while the former was more abundant in roots, meristems, and young leaves, the latter showed higher abundance in the 30DAF and 90DAF perisperms. Notably, *CaGIF2* and *CaGIF5*, genes that clustered in clade I alongside the well-characterized *A. thaliana AtGIF1/AN3*, also displayed specific profiles, with prominent transcript abundance in reproductive and developing tissues, such as flower buds, embryos and the 30DAF perisperm.

### 2.4. CaGRFs and CaGIFs Exhibit Tissue-Specific and Stage-Dependent Transcriptional Shifts in the Starlet Phenotype

**Figure 4:**
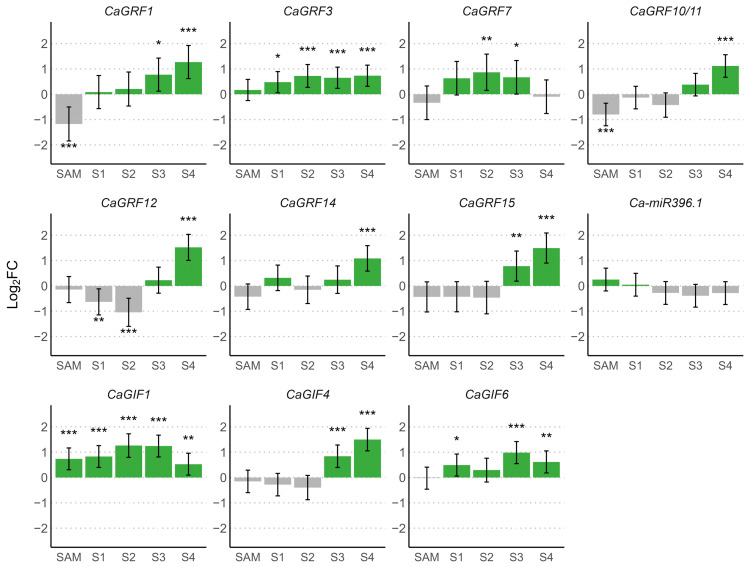
Relative transcript accumulation of *CaGRFs*, *CaGIFs*, and *Ca-miR396.1*. Expression levels were determined via RT-qPCR in shoot apical meristems (SAM) and four developmental stages (S1 to S4) of floral buds collected from typical and starlet phenotypes. Relative expression is presented as log_2_(fold change) calculated using a linear mixed model. Positive values (green bars) indicate up-regulation in the starlet phenotype relative to the normal reference, while negative values (grey bars) denote down-regulation in the starlet plants. Asterisks denote statistical significance: * *p* < 0.01; ** *p* < 0.001; *** *p* < 0.0001. Error bars represent 95% confidence intervals (CI).

Targeted expression analysis by RT-qPCR was performed to evaluate the relative transcript accumulation of selected genes between the typical and starlet phenotypes in SAMs and four developmental stages (S1 to S4) of floral buds (Figure 4). While no consistent expression pattern was observed across gene families during early floral bud development (S1 and S2), a more uniform pattern emerged at intermediate stages (S3 and S4), where all analyzed genes exhibited significantly higher transcript accumulation in starlet samples at one or both stages, with an absence of instances of down-regulation. For instance, *CaGRF1*, *CaGRF15*, and *CaGIF4* were up-regulated at both S3 and S4, while *CaGRF10/11* and *CaGRF14* showed a response restricted to S4. Conversely, *CaGRF7* exhibited an earlier window of activity (S2 and S3), and *CaGIF6* showed a broad but interrupted significant up-regulation (S1, S3, and S4). *CaGRF12* was the sole member to exhibit a biphasic profile, characterized by early down-regulation (S1 and S2), followed by a reversal to up-regulation at S4. Notably, *CaGRF3* and *CaGIF1* were significantly up-regulated across all tissues and developmental stages.

The SAMs of starlet phenotypes exhibited significant down-regulation of *CaGRF1* and *CaGRF10/11*, relative to those of typical plants. In contrast, *CaGIF1* was significantly up-regulated in the starlet samples, while no significant differences in transcript levels were observed for the remaining identified genes. Interestingly, although *CaGRF* transcript levels varied, the expression of *Ca-miR396.1* remained stable across the developmental stages and phenotypes.

## 3. Discussion

### 3.1. Ancestral Expansions and Subgenomic Organization Shape the GRF–GIF Landscape in C. arabica

Growth-Regulating Factors are a family of plant-specific TFs believed to have originated before the emergence of multicellular green plants, within the Charophyte group of freshwater green algae [37]. Nevertheless, it was within the course of evolution of the land plants (Embryophyta) that this gene family expanded substantially, as a result of the multiple whole-genome duplication (WGD) events that are known to have occurred in the group [1]. Through processes of neo- and subfunctionalization, the multiple copies of *GRFs* that have been retained in the genomes of plants now play integral roles in various other aspects of plant development, particularly within the Angiosperms, which usually harbor more than 8 family members [1]. Consistent with this, fifteen *GRFs* were identified in *C. arabica* and their proteins distributed across six major clades that pre-dated the monocot–eudicot divergence (Figure 1). In contrast, the GRF-Interacting Factors constitute a more conserved lineage of transcriptional coactivators with homologs identified across diverse lineages, including humans, underscoring their fundamental role in the eukaryotic transcriptional machinery [15]. Correspondingly, six *GIFs* were identified in the *C. arabica* genome and their proteins categorized into the family’s two major clades (Figure 1). The identification of a smaller number of *CaGIFs* relative to *CaGRFs* is consistent with other plant species and aligns with reports demonstrating the ability of individual GIFs to interact with multiple GRF partners, enabling the assembly of diverse transcriptional modules [13,17].

*C. arabica* is a relatively recent allotetraploid species originated from a single hybridization event between the closely related *C. canephora* and *C. eugenioides* species [27,28]. Thus, *C. arabica* possesses two complete sets of chromosomes with low gene sequence divergence from one another [38], as evidenced by the pronounced collinearity among the two subgenomes (Figure 2). This genomic conservation is reflected in our findings, where most of the identified genes have a corresponding homeolog with similar phylogenetic clustering (Figure 1), structure (Figures S2–S5), subgenome localization (Figure 2), and patterns of expression (Figure 3), suggesting a shared ancestral origin. *CaGRF1* is the only exception, as it is uniquely derived from the parental *C. canephora* subgenome and lacks a clear homeolog from the *C. eugenioides* subgenome. This gene may have arisen from a lineage-specific duplication, differential gene loss, or post-polyploidization genomic restructuring in *C. arabica* [39,40]. Nonetheless, the fact that *CaGRF1* is the sole representative of clade IV, coupled with its distinct expression profile, points toward a specialized, subgenome-specific functional role. Interestingly, our analyses also revealed a subgenome-biased gene expansion within clade III, at the initial portion of chromosome 6C. While the *C. eugenioides* subgenome retained a single member (*CaGRF8*), the *C. canephora* subgenome exhibits three identical copies (*CaGRF4*, *CaGRF5*, and *CaGRF6*), indicating a localized gene duplication event. Genome-wide studies in *C. arabica* have indeed reported that structural chromosomal aberrations, including large-scale duplications, act as a mechanism generating genetic diversity in this recently formed polyploid [40]. Furthermore, our expression data highlight a trend of functional silencing associated with this gene redundancy: transcripts for the three *canephora*-derived copies were undetectable, whereas their *eugenioides*-derived homeolog (*CaGRF8*) maintained a detectable, albeit low, expression level. This asymmetric silencing corroborates the hypothesis of *eugenioides* subgenome dominance previously proposed for *C. arabica*, wherein homeolog silencing exhibits a marked preference against the *C. canephora* subgenome [39].

### 3.2. Phylogenetics and Transcriptomics Implicate the CaGRF–GIF Module in the Starlet Phenomenon

While GRFs are conventionally associated with functional redundancy in the regulation of cell proliferation [21,41], certain family members have acquired specialized, context-dependent roles that contribute to diverse aspects of plant development [2,42]. Although the degree of functional conservation remains to be experimentally confirmed in coffee, inferences from homology-based comparisons provide useful hypotheses regarding the roles of the *CaGRFs* in the starlet phenotype. For instance, our phylogenetic analysis (Figure 1) positioned CaGRF7 in Clade V, grouping them closely with AtGRF5. In *A. thaliana*, AtGRF5 acts as an integrator of cytokinin signaling and leaf development, and its overexpression has been documented to increase chloroplast division and delay leaf senescence [43]. The observation that *CaGRF7* is more expressed in starlet floral buds from S1 to S3 stages (significantly at S2 and S3) (Figure 4) may be associated with the characteristic green coloration of the anomalous flowers, delaying the typical maturation-induced chlorophyll degradation in tissues where that would normally occur. In another example, AtGRF7 (Clade IV) deviates from the typical role of GRFs as transcriptional activators by acting as a repressor of Dehydration-Responsive Element Binding Protein 2a (*DREB2A*) and other osmotic stress-responsive genes, possibly preventing the growth impacts associated with stress response [44]. CaGRF1 was also positioned within Clade IV, yet it exhibited significantly lower transcript levels in the SAMs of starlet plants. This reduction in a putative transcriptional repressor could lead to the de-repression of stress-responsive pathways within the meristematic tissue to the detriment of normal growth, potentially contributing to the developmental alterations that are characteristic of the starlet phenotype. Accordingly, the starlet-flowers are often associated with water deficit events [25,34,36]. Lastly, CaGRF3 was grouped in Clade II alongside AtGRF9, documented to negatively affect organ growth by altering the rate or duration of cell proliferation [45]. The sustained up-regulation of *CaGRF3* across all analyzed stages of starlet-flower bud development suggests that its putative role as a growth repressor may be associated with the restricted development and malformation of the petals in this phenotype. Both proteins share a distinct structural characteristic: the presence of two WRC domains; however, whether this is directly responsible for the repressive activity observed in Clade II members remains to be functionally explored. While these homology-based interpretations remain hypothetical, the correlation between clade-specific expression shifts and the observed morphological anomalies provides a strong basis for future functional validation.

The patterns observed in the coactivator family are also noteworthy. *CaGIF2* and *CaGIF5*, the closest homologs to the canonical *AtGIF1/AN3*, exhibited dynamic and tissue-specific expression profiles, with prominent transcript abundance in reproductive and developing tissues such as flower buds, embryos, and the 30 DAF perisperm (Figure 3). Conversely, the more ubiquitous abundance of the *CaGIF3/CaGIF6* homeologous pair across nearly all analyzed libraries suggests that these Clade II members have assumed the primary roles as constitutive transcriptional coactivators in *C. arabica*. Our RT-qPCR analysis further implicates the coactivator family in the etiology of the starlet phenotype through the significant up-regulation of the three analyzed genes, *CaGIF1*, *CaGIF4*, and *CaGIF6*, although with distinct temporal dynamics (Figure 4). Notably, despite being positioned on homeologous chromosomes (2C and 2E, respectively), the simultaneous, sustained up-regulation of *CaGIF1* and *CaGRF3* through all stages of floral bud development could suggest a coordinated activation across the *C. canephora* and *C. eugenioides* subgenomes. Interestingly, the starlet anomaly was also reported in the diploid *C. canephora* by field observations made by our team (requiring further investigation), although to a much lesser extent than in *C. arabica*. This phenotypic manifestation is a far more prevalent characteristic of the allotetraploid species, where inter-subgenomic crosstalk may occur. Nevertheless, the specific interaction networks between identified CaGIFs and CaGRFs remain undetermined; thus, it is unclear which specific pairings occur in vivo.

### 3.3. A Multilayered Regulation of the CaGRF–GIF Module in the Starlet Phenomenon

Beyond their role in GRF-GIF modules, GIFs are essential for recruiting SWI/SNF chromatin-remodeling complexes to modulate promoter accessibility [19]. Accordingly, an upregulation of the AN3-SWI/SNF module’s activity in *A. thaliana* is implicated in prolonging the exit from the mitotic cell cycle, consequently delaying cell differentiation [19]. Hence, it is plausible that the observed transcriptional shifts might be associated with changes in chromatin state, including DNA methylation, which may affect the regulatory balance required for normal floral development. This potential epigenetic layer and its relationship with the starlet phenomenon represent an intriguing frontier for future functional exploration.

Altogether, our results reveal a stage-dependent reconfiguration of the GRF–GIF transcriptional module in starlet tissues. Differential expression of these regulators in both SAMs and developing floral buds suggests an association with altered developmental trajectories. The presence of transcriptional shifts in the SAM further raises the possibility that the starlet phenotype may involve early alterations in meristem function, prior to floral induction. Alternatively, the phenotype may result from systemic regulatory changes affecting multiple developmental stages. The levels of *Ca-miR396.1* remained stable, suggesting that the observed differences in *CaGRF* abundance in the starlet phenotype are likely not driven by this specific microRNA. However, given that *C. arabica* harbors nine additional distinct mature miR396 molecules [46], the potential contribution of other family members to *CaGRF* transcript accumulation cannot be ruled out. Moreover, the conserved role of these modules as transcriptional hubs integrating environmental signals with developmental programs, supported by the diverse array of putative hormone- and stress-responsive CREs identified within their promoter regions, leads us to hypothesize a conditional transcriptional activation that could outweigh the canonical microRNA-dependent suppression mechanism. While the link between environmental stress and the starlet phenotype still requires experimental validation, such a mechanism would offer a plausible explanation for the sustained accumulation of transcripts observed in our analyses. Despite the relatively modest changes in the expression of each individual gene, redundancy may exert an additive effect, further disrupting the balance of GRF-GIF and downstream targets activities required for proper floral organ development.

## 4. Materials and Methods

### 4.1. Plant Materials

Plant material was obtained from six-year-old *C. arabica* cv. Pau Brasil genotypes grown at the Inova Café experimental field (Federal University of Lavras; 21°23′ S, 44°97′ W, Brazil). The trees were arranged in three randomly distributed field plots and cultivated under nutritional and pest management conditions, as described by Vieira et al. [47]. Daily maximum temperature and total precipitation data were retrieved for the UFLA weather station (ID: 83687; −21.23, −44.98) from the Instituto Nacional de Meteorologia (INMET) database (https://bdmep.inmet.gov.br/, accessed on 27 April 2026) to characterize the environmental context of the study site (Figure S6). Individual trees exhibiting both typical and starlet-flowers simultaneously were selected, and sampling was conducted longitudinally between February and May 2020, at the beginning of the floral development period [48], with more advanced developmental stages harvested from the same monitored plants over time. The experimental design comprised four biological replicates per phenotype and developmental stage, with each biological replicate consisting of a pooled sample from three independent plants. Collected tissues encompassed shoot apical meristems (SAMs) and floral buds at four developmental stages (S1–S4), following the classification by Ribeiro et al. [46]. Briefly, these stages were defined as follows: S1 (buds < 2 mm, undifferentiated); S2 (floral buds < 3 mm); S3 (floral buds 3–6 mm); and S4 (floral buds ~8 mm). Immediately after sampling, tissues were flash-frozen in liquid nitrogen, then homogenized and stored in an ultrafreezer at −80 °C until subsequent analysis.

### 4.2. In Silico Analyses

#### 4.2.1. Gene Identification, Structural and Promoter Analyses

Predicted protein sequences of *Coffea arabica* L. were retrieved from its genome assembly and proteome annotation available at the National Center for Biotechnology Information’s webpage (NCBI, RefSeq assembly GCF_036785885.1), and used in the generation of a local database. Next, downloads were retrieved, from Uniprot [49], Genbank [50], Brassicaceae Database (BRAD) [51], and Sol Genomics [52] databases, of validated GRF and GIF protein sequences of *A. thaliana*, *O. sativa*, *Glycine max* (L.) Merr., *Brassica napus* L., and *S. lycopersicum*. To identify the relevant proteins within the coffee genome, downloaded GRF and GIF sequences were utilized in separate alignments against the proteome of *C. arabica* using BLASTp v2.10.1 [53], with an E-value cutoff of 10^−3^. Concurrently, profile Hidden Markov Model (HMM) searches were performed using the HMMER v3.4 [54] software package with an E-value threshold of 10^−5^. Profile HMMs for the family-specific conserved domains, specifically the QLQ (PF08880) and WRC (PF08879) domains for GRFs and the SNH domain (PF05030) for GIFs, were retrieved from the Uniprot database. Proteins exhibiting a sequence length differing by more than 70% from the mean length of the reference sequences were excluded from subsequent analyses to remove truncated or excessively divergent candidates. Remaining sequences’ additional isoforms were manually removed so that only one protein per genetic locus remained. Lastly, candidate proteins were analyzed for the presence of the characteristic conserved domains (Figure S1) using InterProScan [55] to confirm whether the annotated genes constitute members of the GRF and GIF families with canonical functions. Nomenclature was assigned sequentially from chromosome 1 to 11 according to physical gene position and subgenomic distribution (C and E).

To analyze gene structures, the exon–intron architectures of identified genes were obtained from the GFF3 genome annotation file and visualized using Gene Structure Display Server (GSDS) v2.0 [56] to map the distribution patterns, numbers, and lengths of exons and introns for all family members (Figure S2). Conserved motif analysis of validated CaGRF and CaGIF protein sequences was performed using the Multiple Em for Motif Elicitation (MEME) suite v5.5.9 [57] (Figures S3 and S4). Motif discovery parameters were configured as “zero or one occurrence per sequence (zoops)” mode, with the maximum number of motifs to find set to 10 and a motif width between 6 and 50 amino acids.

To investigate the promoter regions of identified genes for putative *cis*-regulatory elements (CREs), genomic sequences extending 2.6 kb upstream of the transcription start site (TSS) of each gene were retrieved from the reference genome FASTA file using TBtools v2.485 [58]. The sequences were screened and potential CREs identified through searches in the PlantCARE database [59]. Downstream data manipulation of the PlantCARE outputs and the final visualization of the promoter architectures (Figure S5) were performed in R v4.4.0 [60] utilizing packages included in the Tidyverse collection v1.3.0 [61].

#### 4.2.2. Phylogenetic Analyses

To phylogenetically analyze the identified *C. arabica* genes, we first performed a global alignment of their encoded proteins with the curated sequences used previously as references, using the MAFFT v7.475 program [62]. The Jalview v2.11.2.5 [63] software was used for the visualization of the alignments (Figure S1). Phylogenetic trees were generated using the Maximum Likelihood (ML) method implemented in IQ-TREE v3.13 [64]. The best-fit amino acid substitution models were automatically selected by ModelFinder [65] within IQ-TREE based on the Bayesian Information Criterion (BIC). Specifically, the JTTDCMut + F + R5 model was identified as the best-fit evolutionary model for the GRF protein dataset, whereas the JTT + F + I + G4 model was selected for the GIF dataset. Node support was evaluated using 1000 Ultrafast Bootstrap [66] replicates. The resulting phylogenetic trees were rooted using the midpoint method and visualized using FigTree v1.4.4 (http://tree.bio.ed.ac.uk/software/figtree/, accessed on 26 March 2026) (Figure 1A,B). Due to the absolute sequence identity shared among CaGRF4, CaGRF5, and CaGRF6, these proteins were merged and are denoted as CaGRF4/5/6 within the phylogenetic tree.

#### 4.2.3. Syntenic Analysis and Chromosomal Mapping

Self-synteny analysis was conducted with SyMap v5.4.6 [67]. GFF and genome sequence files obtained from *C. arabica’s* NCBI annotation were converted into the input format required using SyMap’s ConvertNCBI script, and the analysis was performed using the program’s standard parameters. Location data corresponding to each *CaGRF* and *CaGIF* was obtained from the GFF3 annotation file. Circos v0.69.9 [68] was used for the creation of Figure 2.

#### 4.2.4. RNA-Seq Analyses

To verify the transcriptional activity of *CaGRFs* and *CaGIFs*, expression profiles were determined through analyses of validated RNA-seq libraries of *C. arabica* publicly available in the Sequence Read Archive (SRA) database [69]. Prior to analysis, libraries went through a thorough assessment of quality using FastQC v0.12 (https://www.bioinformatics.babraham.ac.uk/projects/fastqc/, accessed on 26 March 2026) and were trimmed, as necessary, with Trimmomatic v0.39 [70]. Accession numbers and further information on the respective libraries are provided in Table S1. Expression analysis involved several steps. Initially, fragment alignments were carried out against the *C. arabica* genome using the STAR program, version 2.7.8a [71] with standard parameters. Next, the processed libraries were sorted and duplicate reads were removed with the Picard tool v3.4 [72], and the number of mapped fragments was determined using the htseq-count algorithm [73], with the non-unique = all parameter, to account for the high sequence similarity between *C. arabica* homeologous subgenomes and prevent the underestimation of expression for highly conserved gene pairs. Downstream processing of read counts were performed using the edgeR package [74]. Low-abundance genes with a mean raw count < 5 were excluded to filter out background noise, and normalization factors were calculated using the Trimmed Mean of M-values (TMM) method [75] to eliminate library composition biases. Normalized expression levels were subsequently calculated as Fragments Per Kilobase of transcript per Million mapped reads (FPKM). For the visualization of expression profiles, FPKM values were transformed to a logarithmic scale (log_10_(FPKM + 1)), and heatmaps (Figure 3A,B) were generated using the pheatmap package [76].

#### 4.2.5. microRNA Selection

In order predict the coffee tree’s miRNA molecules with the potential to target the identified *CaGRFs*, we subjected the sequences of the transcripts of these genes to an analysis using the psRNATarget tool [77], with standard parameters, where we also provided all non-redundant mature miRNA sequences identified in *C. arabica* [46]. Based on the analysis, the miR396.1.abcd sequence, referred to here as Ca-miR396.1, was chosen for subsequent gene expression analyses, as it is expected to target all *CaGRFs* (Table S1).

### 4.3. Gene Expression Analyses

#### 4.3.1. Primer Design

Primers for Reverse Transcription quantitative Polymerase Chain Reaction (RT-qPCR) were designed specifically for selected *CaGRFs* and *CaGIFs* with the Primer-BLAST [78] tool, following the recommended specifications for qPCR [79]. Candidate oligos were examined with OligoAnalyzer v3.1 [80] for the inspection of primer parameters and possible hairpin or dimer formations. Primers for stem-loop RT-PCR were designed specifically for the *Ca-miR396.1* according to Varkonyi-Gasic et al. [81]. All primers used are listed in Table S1.

#### 4.3.2. RNA Extraction, DNAse Treatment and cDNA Synthesis

Total RNA extraction was conducted according to the protocol established by Oliveira et al. [82]. The extracted RNA samples were analyzed by a spectrophotometer (*GE NanoVue*™, GE Healthcare, Chicago, IL, USA) with the purpose of ensuring good molecule concentration and quality. Residual DNA was removed with TURBO DNA-free™ Kit (Thermo Fisher, Waltham, MA, USA), and the treated RNAs were subsequently assessed for their integrity via electrophoresis on a 1% agarose gel with GelRed Nucleic Acid Gel Stain dye (Sigma-Aldrich, Burlington, MA, USA). The complementary DNA (cDNA) of target genes was synthesized using a High-Capacity cDNA Reverse Transcriptase Kit (Thermo Fisher). The synthesis of cDNA from the mature miR396.1 molecule was performed using the stem-loop RT-PCR method described by Varkonyi-Gasic et al. [81] with the Improm-II Reverse Transcriptase kit (Promega, Madison, WI, USA). RNA treatment and cDNA synthesis were carried out in a Veriti thermocycler (Applied Biosystems, Waltham, MA, USA).

#### 4.3.3. RT-qPCR

Gene expression analyses by RT-qPCR were conducted in a Rotor-Gene Q Real-Time PCR thermocycler (QIAGEN, Venlo, Netherlands), as described in [83]. Four biological repetitions with two technical replicates each were used per experimental group (phenotype × developmental stage). Candidate genes were prioritized for validation based on the preceding in silico findings, with the purpose of attaining comprehensive insight into the two gene families. Due to the high sequence identity (98.31%) between *CaGRF10* and *CaGRF11*, which precluded the design of gene-specific primers, these transcripts were merged and denoted as *CaGRF10/11*. Furthermore, given the conserved role of the miR396 family in the post-transcriptional regulation of *GRFs*, target prediction analysis was performed (see Section 4.2.5), and *Ca-miR396.1* was included in the RT-qPCR analysis to evaluate potential correlations between the levels of the miRNA and its targets during floral development.

The genes *CaACT* (Actin) and *CaUBQ2* (Ubiquitin-conjugating enzyme E2), described in [84,85,86], were used as references for normalization, given their previous validation across multiple independent experiments, tissues, and experimental conditions in *C. arabica*. Nevertheless, to ensure their reliability within our specific dataset, their expression stability was further evaluated using the BestKeeper tool [87] included in the RefFinder platform [88] (Table S1). Amplification efficiencies for each primer pair were calculated directly by the Rotor-Gene Q series software v2.2.3 from a five-point standard curve generated using a pooled cDNA sample consisting of aliquots from all experimental groups. The efficiencies are provided in Table S1 alongside raw cycle quantification (C_q_) values. Annealing and extension were carried out at 60 °C for 10 s for most targets, while an optimized temperature of 62 °C was applied for *CaGRF7* and *CaGIF1*. Following amplification, a melting curve analysis was performed to confirm single-product amplification and the absence of primer dimers. The melting protocol consisted of a temperature ramp from 55 °C to 95 °C, rising by 1 °C per step, with a 90 s pre-melt conditioning on the first step followed by a 5 s hold per subsequent step, with continuous fluorescence acquisition. Representative dissociation curves for each analyzed gene are displayed in Figure S7.

Expression ratios were calculated as log2-fold changes between the starlet and typical phenotypes at each developmental stage using a Linear Mixed Model, following the methodology outlined by Steibel et al. [89], after correcting raw C_q_ values for calculated primer efficiencies. The model was implemented via the lme4 package v1.1 [90], incorporating sample type and developmental stage as fixed effects and biological replicates as random effects. Post hoc pairwise contrasts were performed using the multcomp package v1.4 [91], with *p*-values adjusted for multiple comparisons using the single-step method.

## 5. Conclusions

Overall, this is the first study specifically focused on the molecular basis of the occurrence of abnormal flowers in *C. arabica*, shedding initial light on the altered genetic pathways underlying the “starlet” phenomenon. Although comprehensive expression profiling of the remaining mature miR396 family members is necessary to confirm whether the CaGRF-CaGIF module operates independently of microRNA-mediated regulation in this phenomenon, future research should also prioritize identifying specific protein-partner affinities within the coffee GRF−GIF network to determine how the distinct pairings contribute to the phenotypic anomaly. Furthermore, given the role of GIFs in recruiting SWI/SNF complexes, characterizing the epigenomic landscape of starlet tissues is an important next step, as the anomaly may be rooted in altered chromatin accessibility or DNA methylation patterns. In parallel, controlled physiological and environmental assays are needed to experimentally validate the relationship between specific climatic variables, the predicted environmental and hormonal regulation of the GRF-GIF module, and the floral anomaly. Finally, elucidating the modules’ downstream targets, in particular those involved in meristem maintenance and floral morphogenesis, remains essential to fully elucidate the physiological and molecular mechanisms associated with this developmental alteration in such an economically significant crop species.

## Data Availability

The original contributions presented in this study are included in the article/Supplementary Materials. Further inquiries can be directed to the corresponding author.

## Supplementary Materials

The following supporting information can be downloaded at https://www.mdpi.com/article/10.3390/ijms27136007/s1.

## Abbreviations

The following abbreviations are used in this manuscript:

GRF	Growth-Regulating Factor
GIF	GRF-Interacting Factor
SAM	Shoot Apical Meristem
TF	Transcription Factor
KNOX	Knotted1-Like Homeobox
SNH	SYT N-terminal homology
BRM	BRAHMA
SWI/SNF	Switch/Sucrose Nonfermenting
AN3	ANGUSTIFOLIA3
CREs	*Cis*-Regulatory Elements
DAF	Days After Flowering
CI	Confidence Interval
WGD	Whole-Genome Duplication
DREB2A	Dehydration-Responsive Element Binding Protein 2a
INMET	Instituto Nacional de Meteorologia
NCBI	National Center for Biotechnology Information
HMM	Hidden Markov Model
GSDS	Gene Structure Display Server
MEME	Multiple Em for Motif Elicitation
TSS	Transcription Start Site
SRA	Sequence Read Archive
TMM	Trimmed Mean of M-values
FPKM	Fragments Per Kilobase of transcript per Million mapped reads
cDNA	Complementary DNA
RT-qPCR	Reverse Transcription–Quantitative Polymerase Chain Reaction
ACT	Actin
UBQ2	Ubiquitin-conjugating enzyme E2
