# Supplementary material for: Genome-Wide Analysis of the GRF-GIF Module in Coffea arabica L.: Insights into the Starlet-Flower Phenomenon
Source: Int J Mol Sci. 2026 Jul 4;27(13):6007. doi: 10.3390/ijms27136007 (PMC13362512; doi:10.3390/ijms27136007)
Supplement: Supplementary file 1 [file ijms-27-06007-s001.zip › supplement_legends.pdf]

Figure S1. Multiple sequence alignment (MSA) of the characteristic conserved domains in CaGRF and CaGIF sequences. (A) Alignment of the typical QLQ and WRC domains across the fifteen identified CaGRF sequences. (B) Alignment of the conserved SNH domain in the six identified CaGIF sequences. Amino acid residues are colored according to their physicochemical properties. Residue positions are indicated above the sequences, while conservation scores (0–9) are displayed as a histogram at the base of each alignment.

Figure S2. Exon–intron structural organization of *CaGRF* and *CaGIF* gene families. (A) Gene structure diagrams for the fifteen *CaGRF* members. (B) Gene structure diagrams for the six *CaGIF* members. Green arrows represent exons, whereas thin black lines represent introns. The 5′ and 3′ relative orientations and gene lengths in base pairs (bp) are displayed along the x-axis.

Figure S3. Distribution of conserved protein motifs within the CaGRF family. The 10 conserved motifs were identified using the MEME suite and are represented by distinct colored boxes numbered 1 to 10. The relative positions of the motifs are mapped along the horizontal lines representing each individual protein sequence, with their corresponding p-values listed on the left. The bottom panel displays the specific color symbols and consensus amino acid sequences for each identified motif.

Figure S4. Distribution of conserved protein motifs within the CaGIF family. The 10 conserved motifs were identified using the MEME suite and are represented by distinct colored boxes numbered 1 to 10. The relative positions of the motifs are mapped along the horizontal lines representing each individual protein sequence, with their corresponding p-values listed on the left. The bottom panel displays the specific color symbols and consensus amino acid sequences for each identified motif.

Figure S5. Predicted *cis*-regulatory elements (CREs) in the promoter regions of (A) *CaGRFs* and (B) *CaGIFs*. Analyzed genomic sequences encompass the 2.6 kb region upstream of the transcription start site (TSS). Colored squares indicate the relative positions of predicted CREs identified via the PlantCARE database. For clarity, motifs were grouped into functional categories: phytohormone responsiveness (ABA, GA, SA, MeJA, and auxin), abiotic stress (drought, low temperature, anaerobiosis, and wounding), seed-specific regulation, and growth-related processes. The x-axis indicates the position in base pairs (bp) relative to the TSS (position 0).

Figure S6. Daily meteorological data of the study site during 2020. The plot displays daily records of total precipitation (mm, blue line) shown on the primary y-axis (left) and maximum temperature (°C, red line) on the secondary y-axis (right), plotted against the months of the year 2020 on the X-axis.

Figure S7. Representative RT-qPCR dissociation curves for analyzed *CaGRFs* and *CaGIFs*, *Ca-miR396.1*, and reference genes (*CaACT* and *CaUBQ2*). Each panel plots the negative derivative of fluorescence with respect to temperature (dF/dT) on the y-axis, as a function of temperature (°C), on the x-axis. The overlapping, colored lines within each chart represent the two technical replicates.

Table S1. Characterization, *in silico* predictions and RT-qPCR experimental parameters for *CaGRF* and *CaGIF* families. First sheet: Gene nomenclature, corresponding *Arabidopsis* homologs, phylogenetic clades, GenBank accession numbers, genomic coordinates, and transcript/protein lengths. Second sheet: Predicted *cis*-

regulatory elements within the 2,600 bp upstream promoter regions identified via PlantCARE. Third sheet: Predicted post-transcriptional interactions between *Ca-miR396.1* and *CaGRF* transcripts generated via psRNATarget analysis. Fourth sheet: Oligonucleotide primer sequences and amplification efficiencies for stem-loop RT-qPCR and conventional RT-qPCR experiments. Fifth sheet: Raw quantification cycle (C_q) values obtained from RT-qPCR assays and reference gene stability evaluation. Subsequent sheets include metadata for the public Sequence Read Archive (SRA) transcriptomic libraries utilized for expression profiling, and a curated list of reference protein sequences from *A. thaliana*, *O. sativa*, *G. max*, *B. napus*, and *S. lycopersicum* utilized for local database generation and phylogenetic reconstruction.
