# Supplementary figures and images for: Genome-Wide Analysis of the GRF-GIF Module in Coffea arabica L.: Insights into the Starlet-Flower Phenomenon
Source: Int J Mol Sci. 2026 Jul 4;27(13):6007. doi: 10.3390/ijms27136007 (PMC13362512; doi:10.3390/ijms27136007)

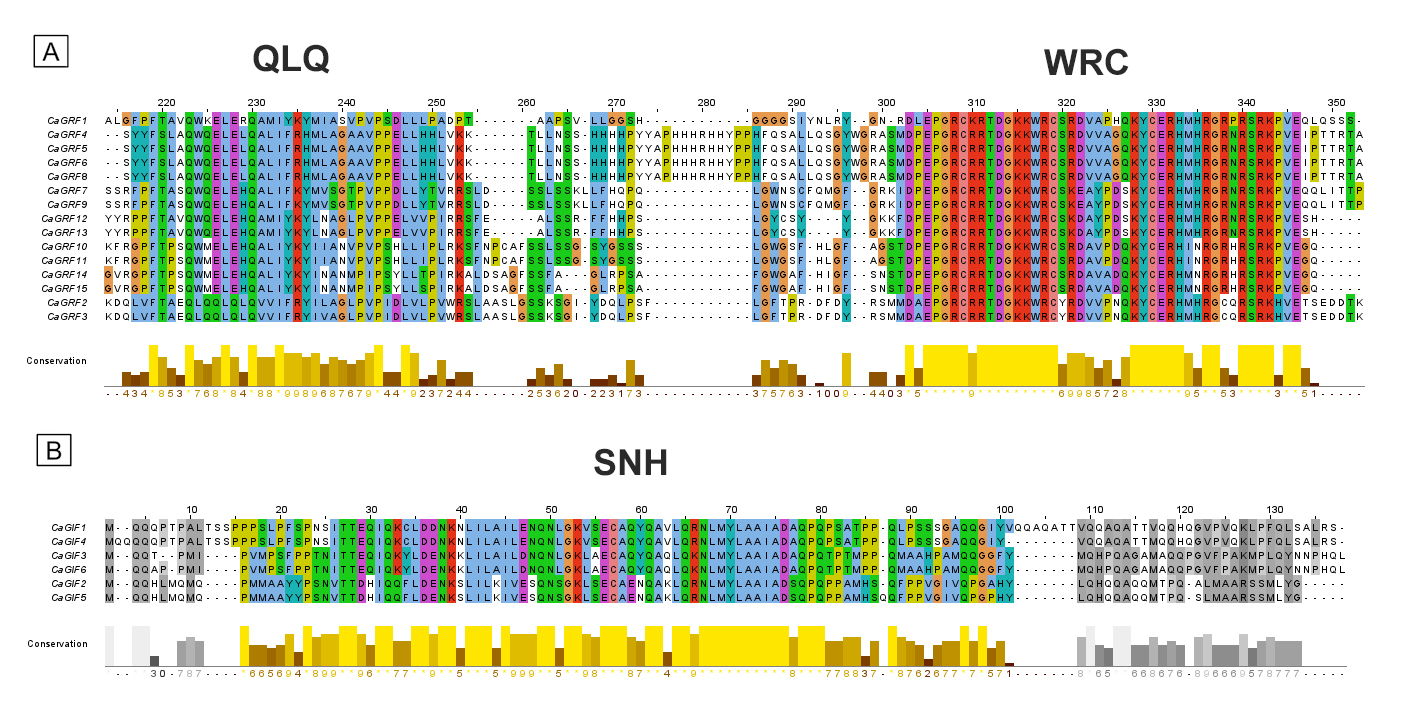

Supplement: Supplementary file 1 [file ijms-27-06007-s001.zip › Figure_S1.jpg]

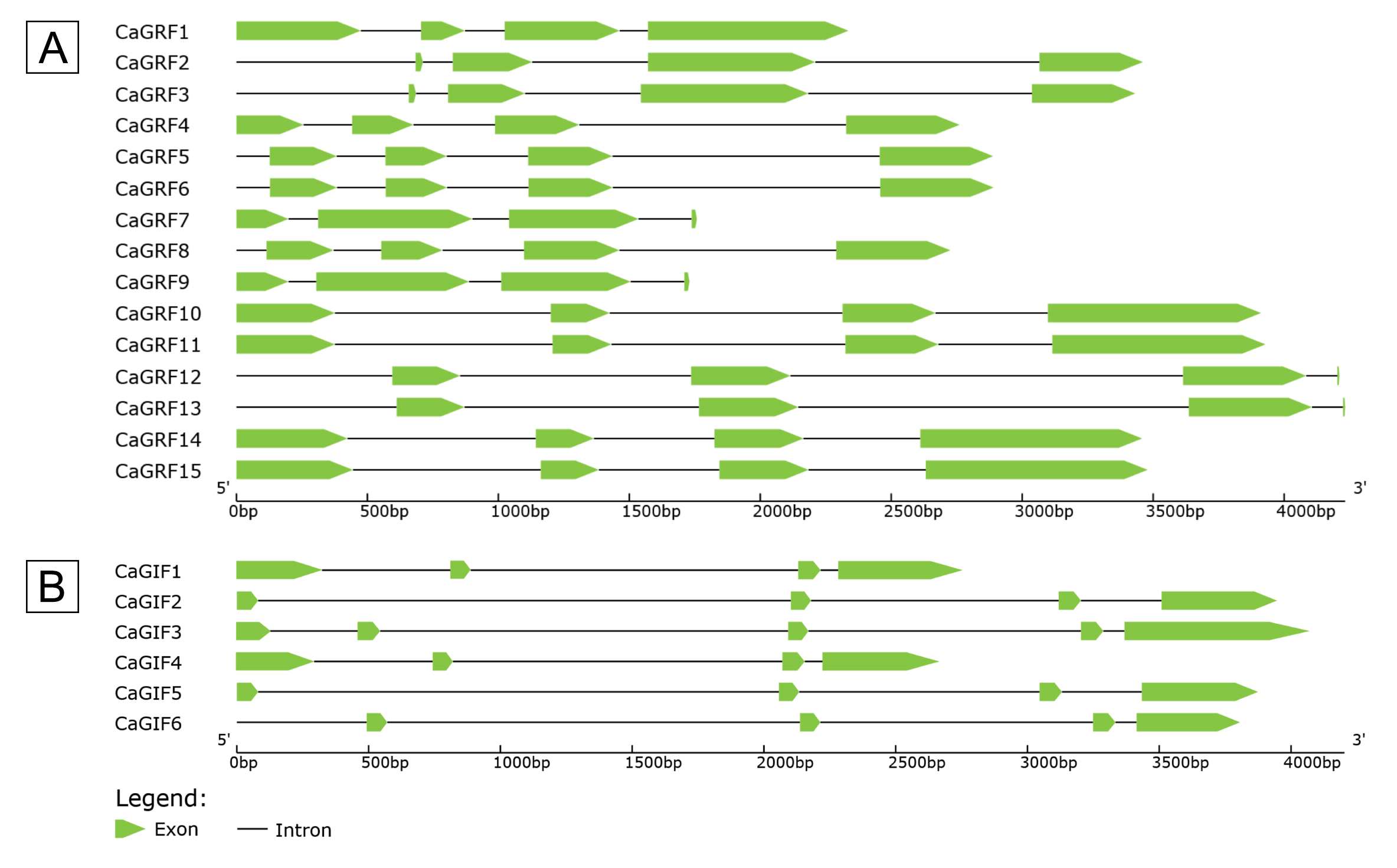

Supplement: Supplementary file 1 [file ijms-27-06007-s001.zip › Figure_S2.jpg]

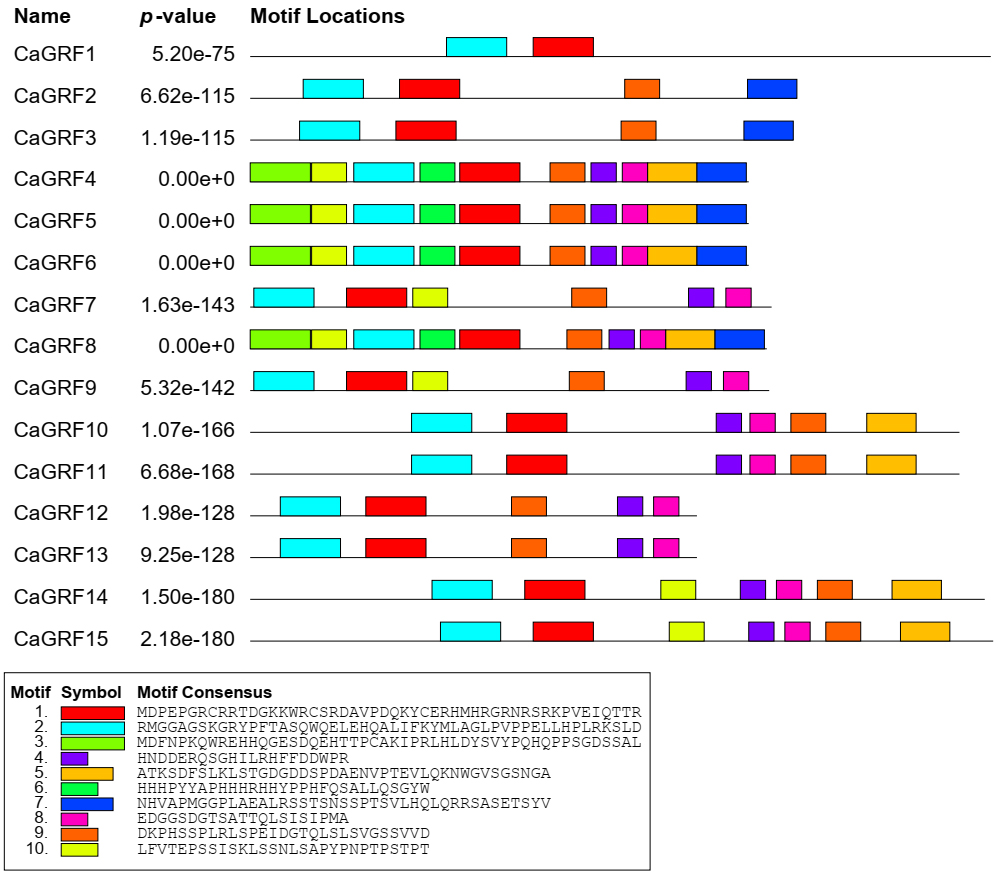

Supplement: Supplementary file 1 [file ijms-27-06007-s001.zip › Figure_S3.jpg]

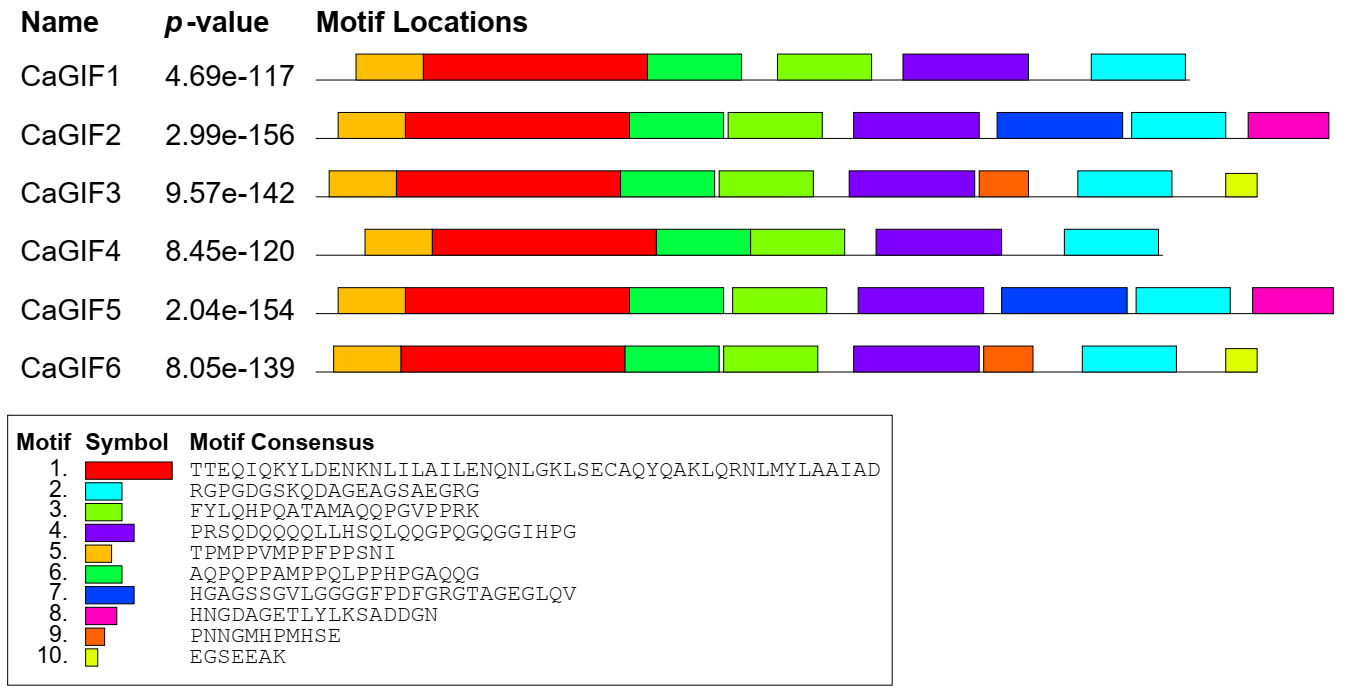

Supplement: Supplementary file 1 [file ijms-27-06007-s001.zip › Figure_S4.jpg]

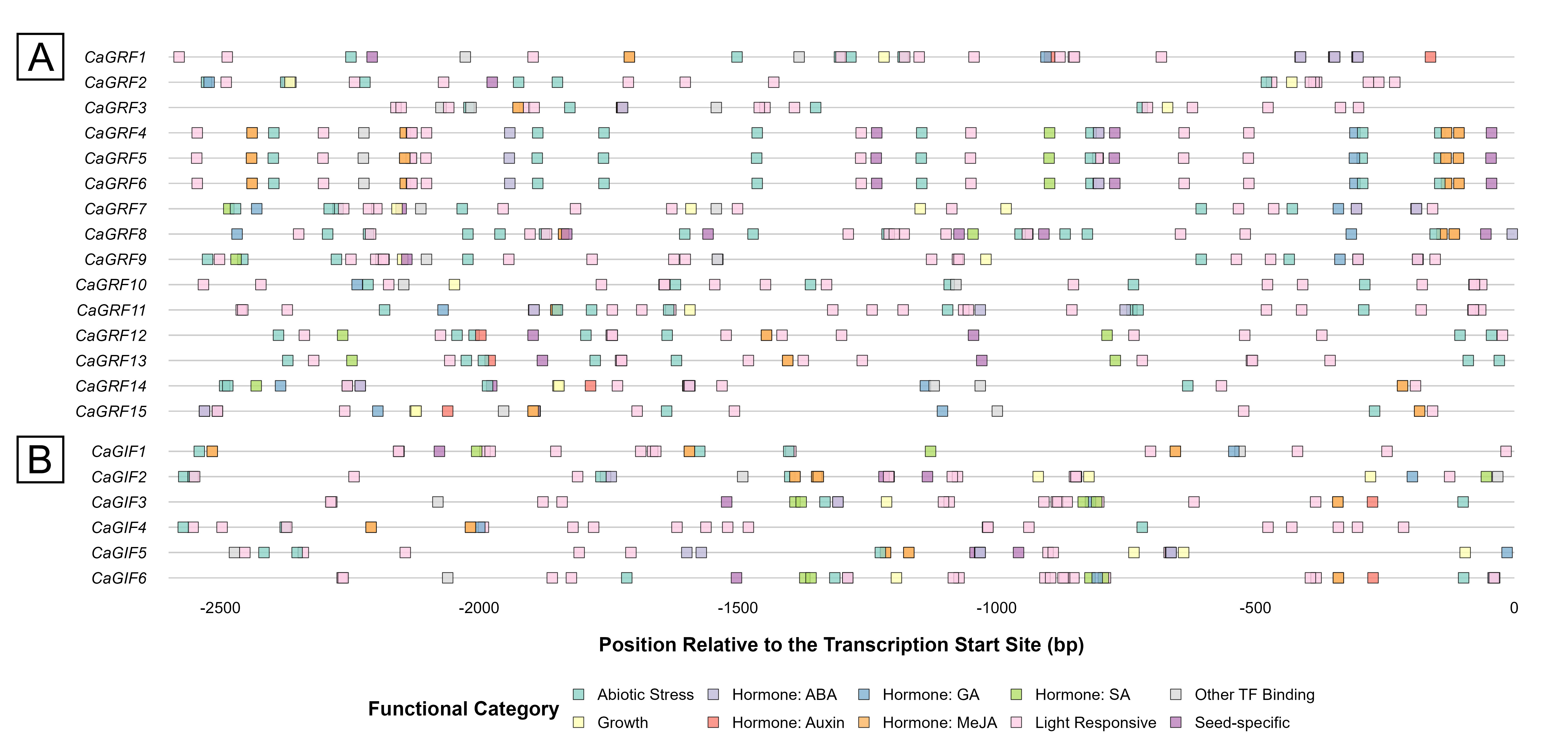

Supplement: Supplementary file 1 [file ijms-27-06007-s001.zip › Figure_S5.jpg]

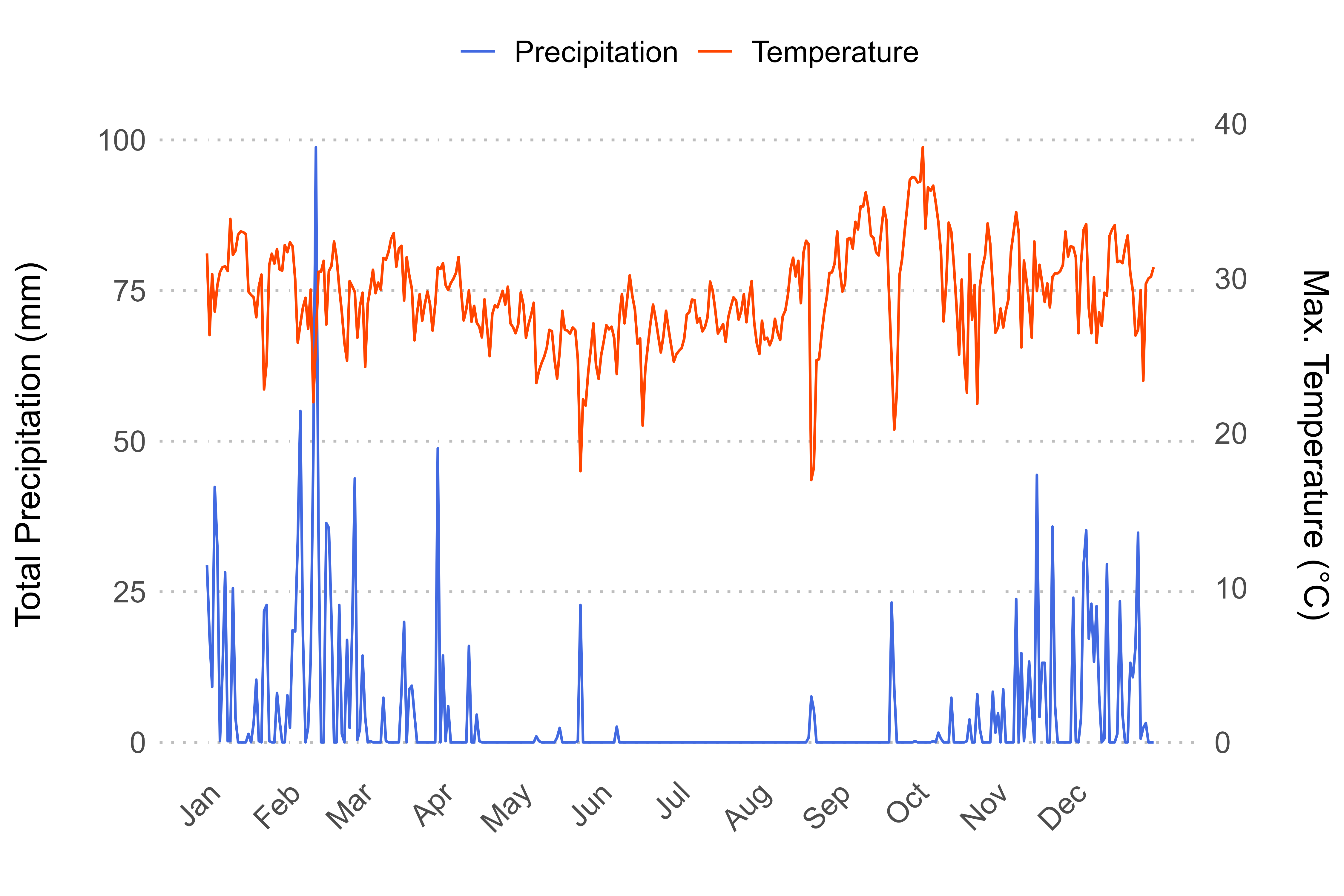

Supplement: Supplementary file 1 [file ijms-27-06007-s001.zip › Figure_S6.png]

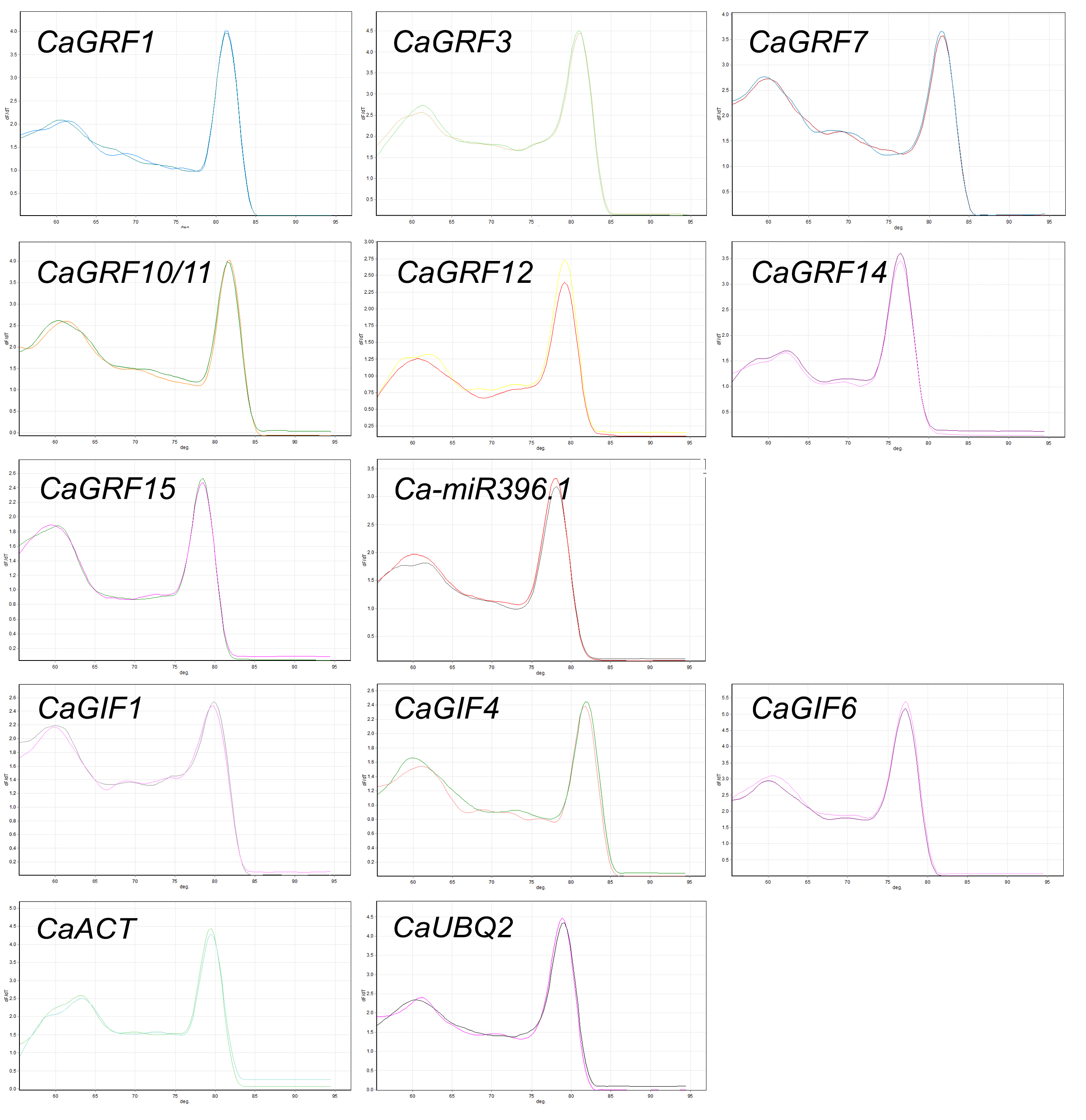

Supplement: Supplementary file 1 [file ijms-27-06007-s001.zip › Figure_S7.jpg]
